# Supplementary material for: p53 suppresses MHC class II presentation by intestinal epithelium to protect against radiation-induced gastrointestinal syndrome
Source: Nat Commun. 2024 Jan 2;15:137. doi: 10.1038/s41467-023-44390-w (PMC10762193; doi:10.1038/s41467-023-44390-w)
Supplement: Supplementary file 1 — Supplementary Information [file 41467_2023_44390_MOESM1_ESM.pdf]

Supplementary Figures and Tables

Supplementary Figure 1

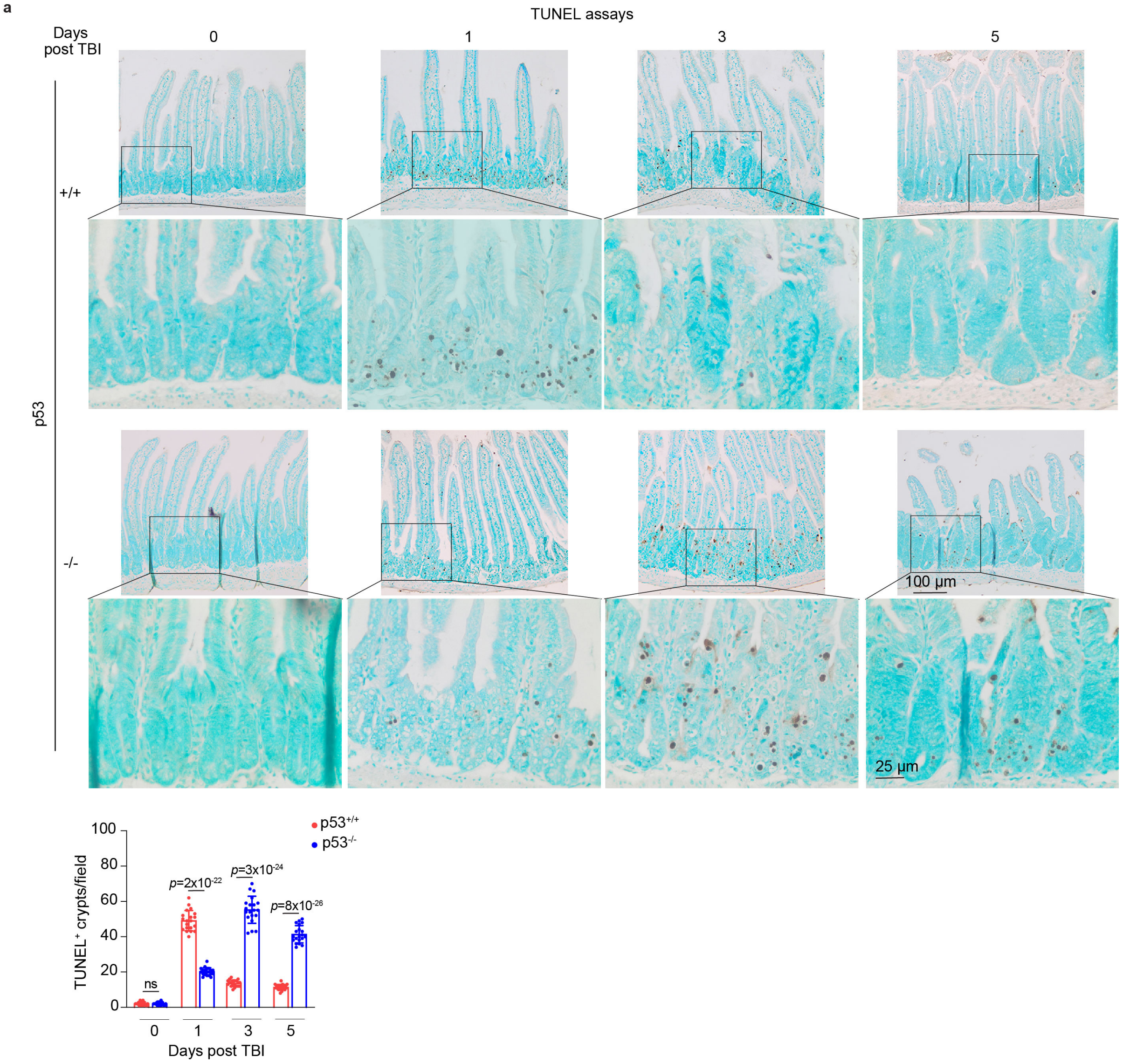

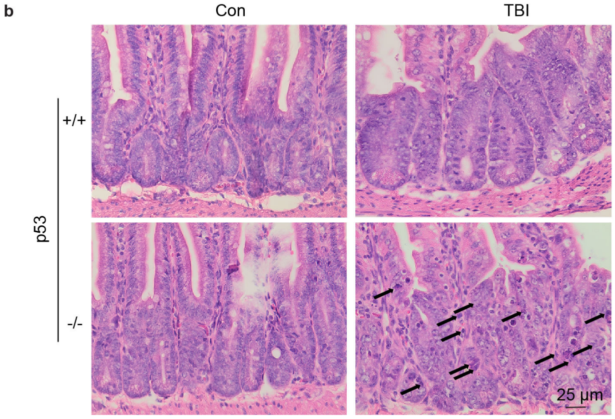

Figure S1. p53 deficiency results in severe cell death in the SI of mice post-TBI.

a. Representative images from at least 3 independent mice (upper panels) and quantification (bottom panel) of TUNEL assays in the SI of $p53^{+/+}$ and $p53^{-/-}$ mice at different time points post 12 Gy TBI. n = 20 fields from at least 3 mice/group. **b**. Representative images from at least 3 independent mice showing mitotic catastrophe in the SI of $p53^{+/+}$ and $p53^{-/-}$ mice at 3 days post 12 Gy TBI. Data are presented as mean ± SD from at least 3 independent experiments. ns: not significant, two-tailed Student's *t*-test. Source data are provided as a Source Data file.

Supplementary Figure 2

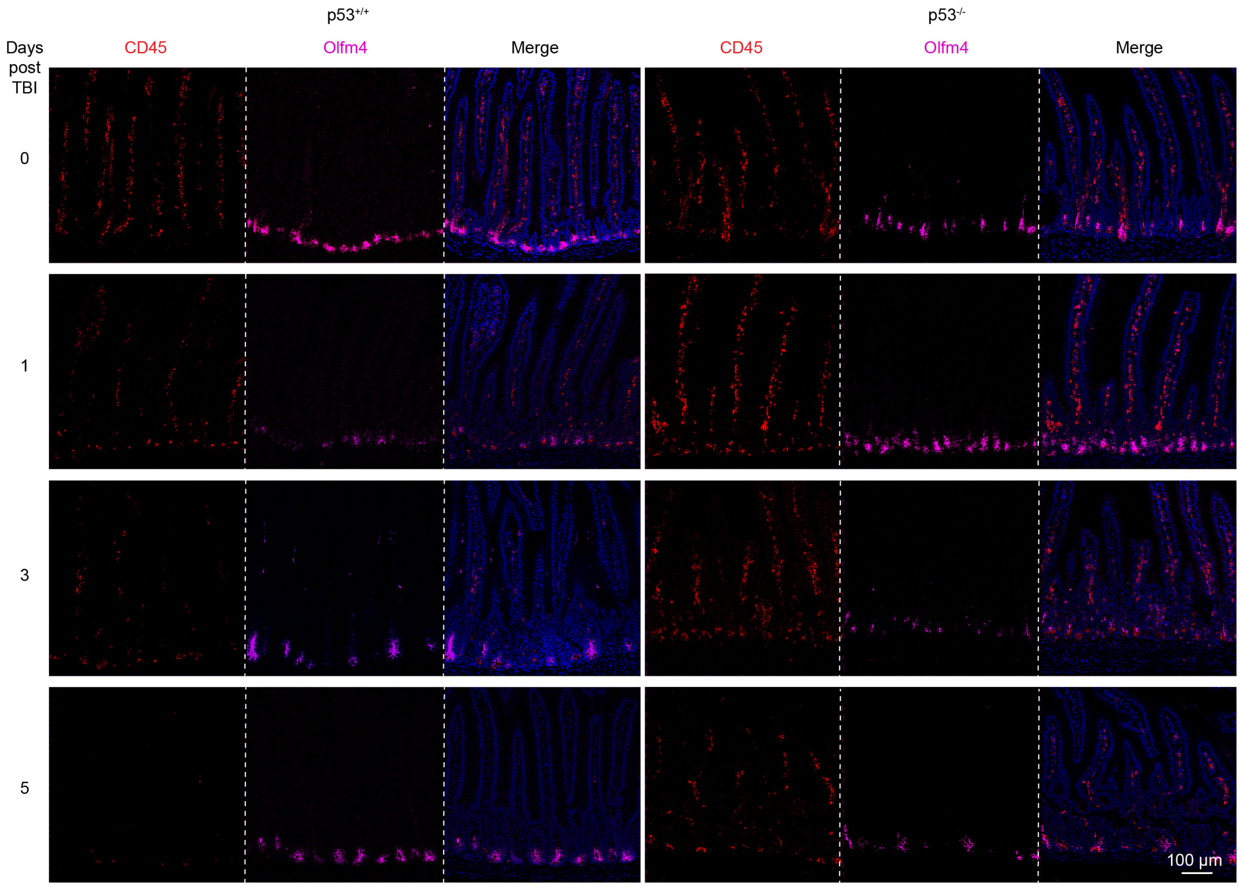

Figure S2. p53 deficiency enhances the inflammatory immune response and ISC damage in the SI of mice post-TBI.

Representative images from at least 3 independent mice showing IF staining of CD45 and Olfm4 in the SI of p53$^{+/+}$ and p53$^{-/-}$ mice at different days post 12 Gy TBI.

Supplementary Figure 3

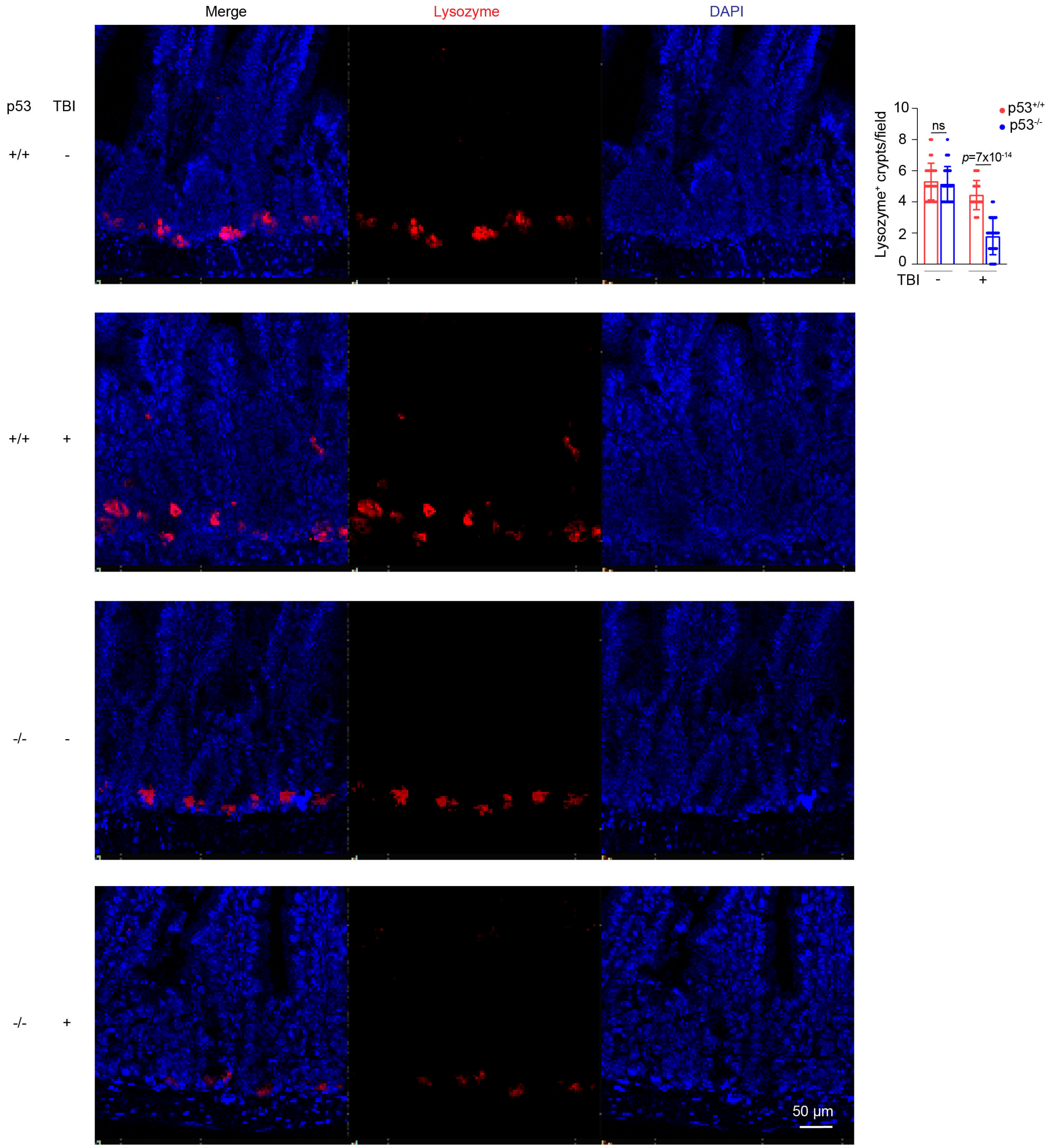

Figure S3. p53 deficiency enhances the damage of Paneth cells in the SI of mice post-TBI. Representative images from at least 3 independent mice (left panels) and quantification (right panel) of IF staining for lysozyme, a Paneth cell marker, in the SI of $p53^{+/+}$ and $p53^{-/-}$ mice at 3 days post-TBI. n = 30 crypts from at least 3 mice/group. Data are presented as mean ± SD from

at least 3 independent experiments. ns: not significant, two-tailed Student's *t*-test. Source data are provided as a Source Data file.

Supplementary Figure 4

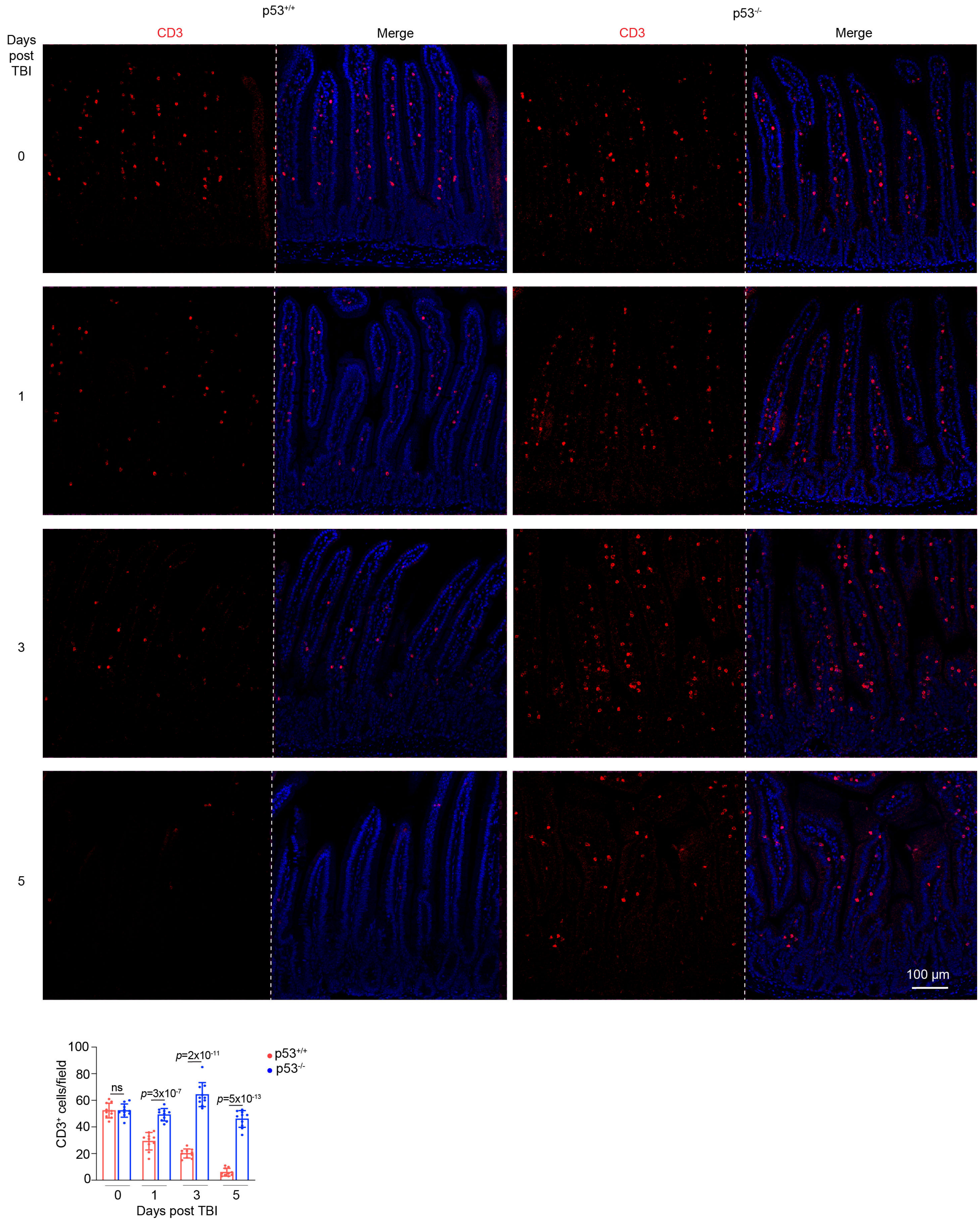

Figure S4. p53 deficiency enhances the number of CD3+ T cells in the SI of mice post-TBI.

Representative images from at least 3 independent mice (upper panels) and quantification (bottom panel) of IF staining of CD3 in the SI of $p53^{+/+}$ and $p53^{-/-}$ mice at different days post-TBI. n = 10 fields from at least 3 mice/group. Data are presented as mean ± SD from at least 3 independent experiments. ns: not significant, two-tailed Student's *t*-test. Source data are provided as a Source Data file.

Supplementary Figure 5

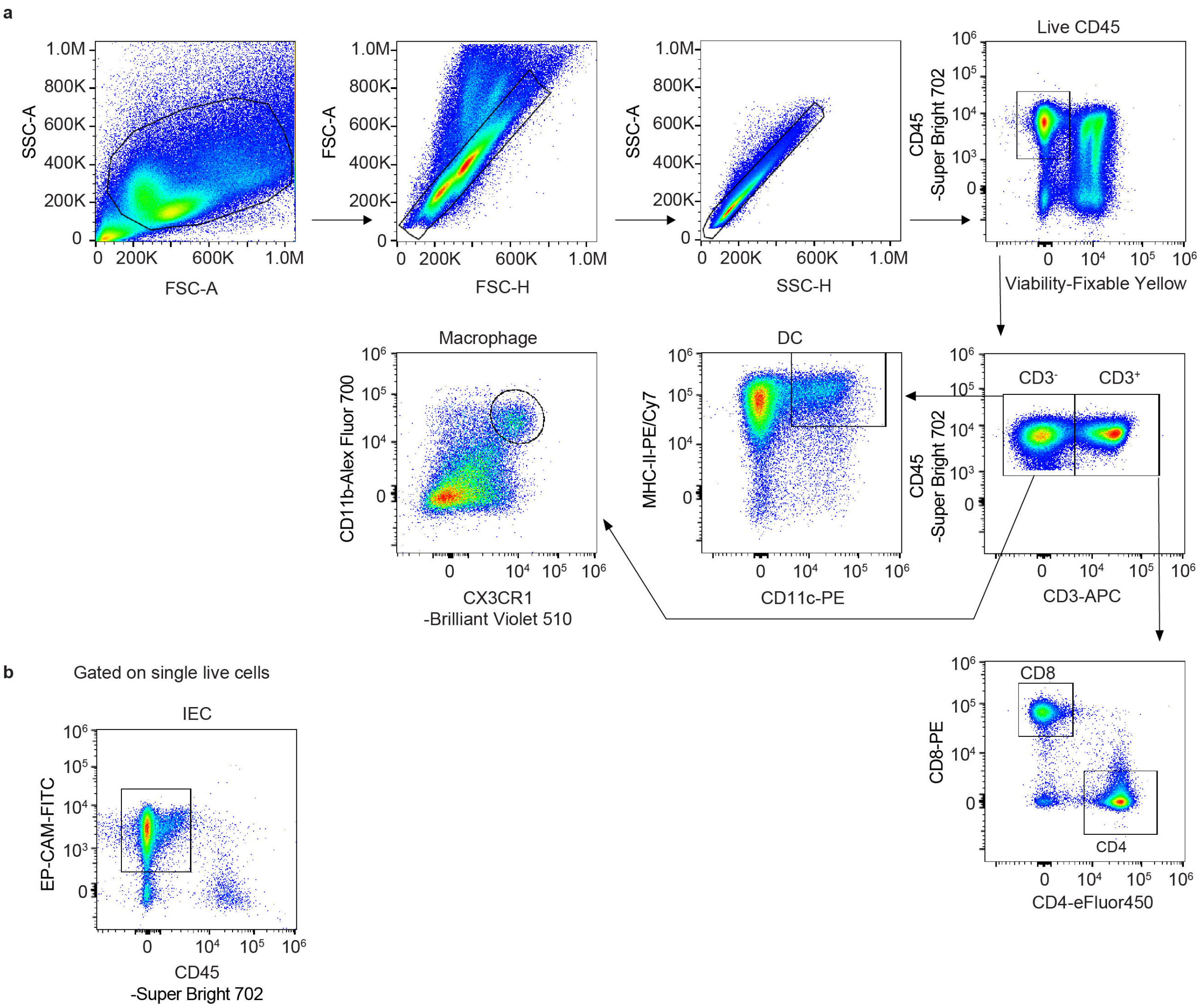

Figure S5. Gating strategies for different immune cell populations and IEC population.

a. Gating strategies for macrophages ($CD45^+CD3^-CX3CR1^+CD11b^+$), DCs ($CD45^+CD3^-$MHC-$II^+CD11c^+$), $CD8^+$ T cells ($CD45^+CD3^+CD8^+$), and $CD4^+$ T cells ($CD45^+CD3^+CD4^+$). **b**. Gating strategies for IECs ($CD45^-EpCAM^+$).

Supplementary Figure 6

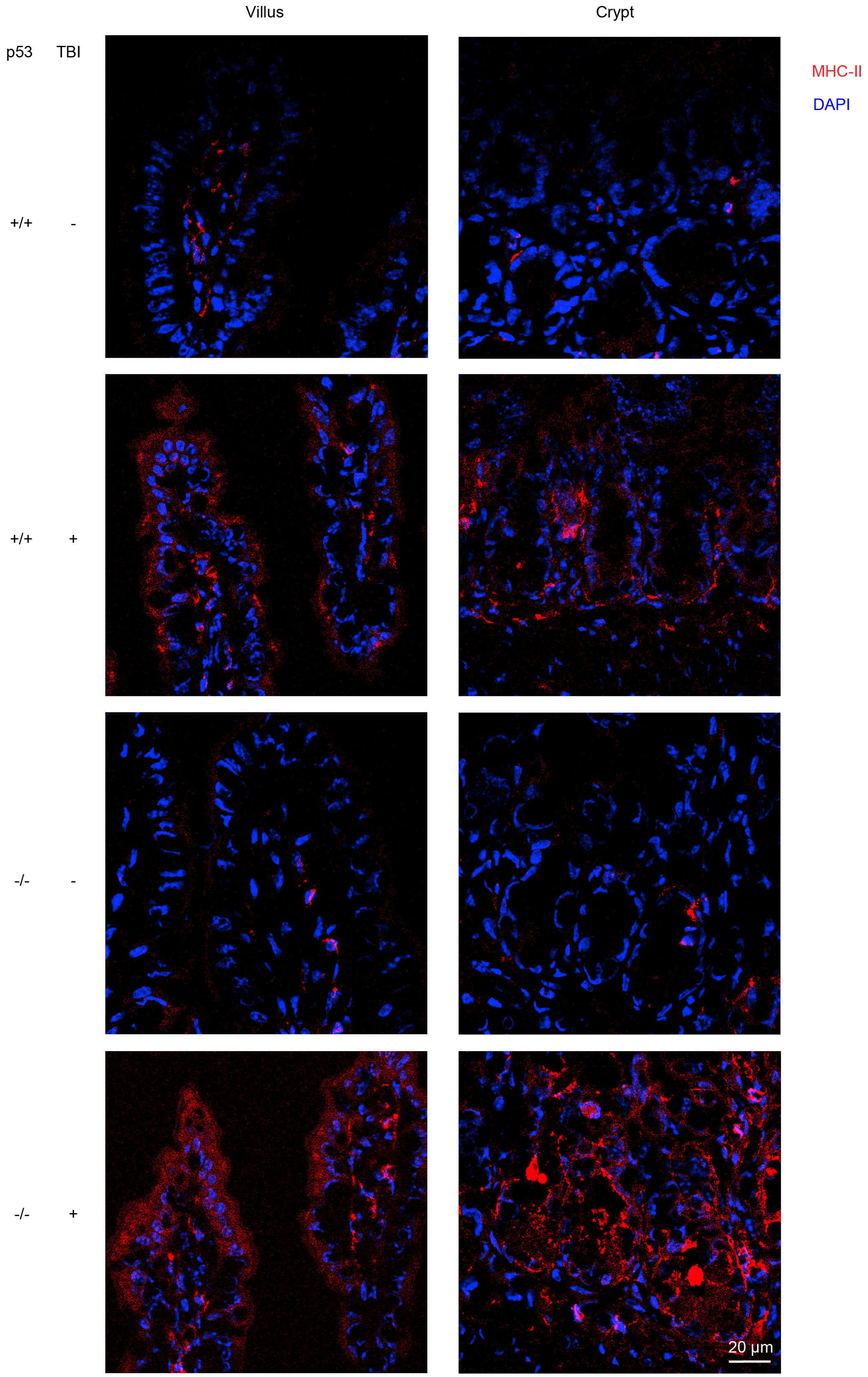

Figure S6. p53 deficiency induces MHC-II expression on both villus and crypt cells in the SI of mice post-TBI.

Representative images from at least 3 independent mice showing IF staining of MHC-II in the SI of $p53^{+/+}$ and $p53^{-/-}$ mice at 3 days post-TBI.

Supplementary Figure 7

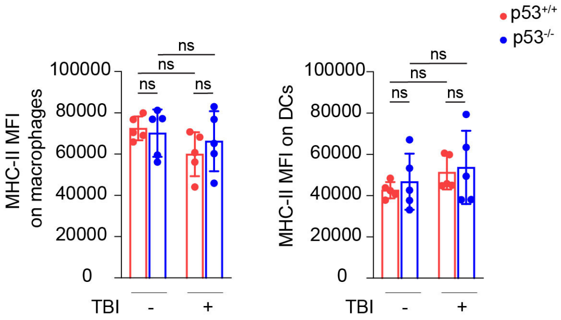

Figure S7. p53 deficiency has no obvious effect on MHC-II expression on macrophages and DCs in the LP of mice post-TBI.

Quantifications of MFI of MHC-II on macrophages (left panel) and DCs (right panel) in the LP of mice at 3 days post-TBI as determined by flow cytometric assays. n = 5 mice/group. Data are presented as mean ± SD from at least 3 independent experiments. ns: not significant, two-tailed Student's *t*-test followed by Bonferroni correction. Source data are provided as a Source Data file.

Supplementary Figure 8

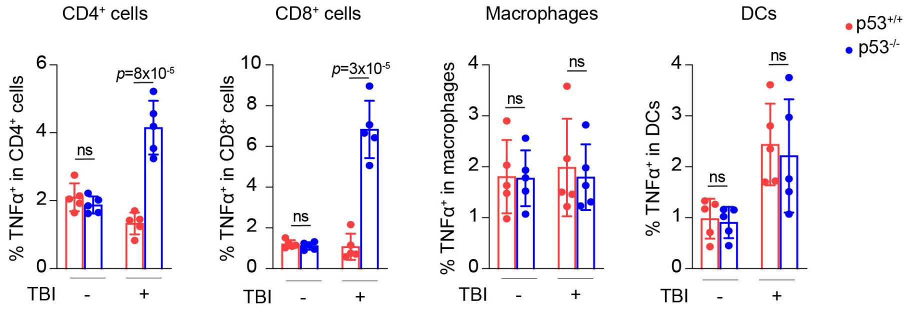

Figure S8. p53 deficiency promotes TNFα secretion from T cells in the LP of mice post-TBI.

Quantification of TNFα secretion from CD4$^+$, CD8$^+$, macrophages and DCs in the LP of mice at 3 days post-TBI as determined by flow cytometric assays. LP cells isolated from mice were stimulated with eBioscience™ Cell Stimulation Cocktail containing PMA, ionomycin, brefeldin A and monensin for 6 hours at 37°C. After cell surface staining, cells were fixed and permeabilized, followed by intracellular staining of TNFα. n = 5 mice/group. Data are presented as mean ± SD from at least 3 independent experiments. ns: not significant, two-tailed Student's *t*-test. Source data are provided as a Source Data file.

Supplementary Figure 9

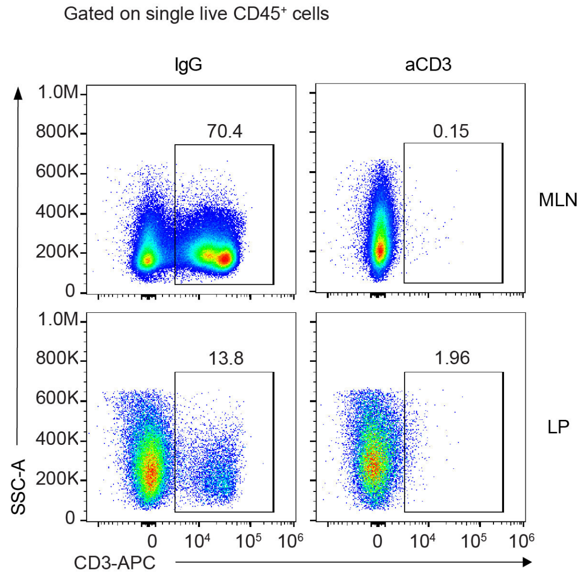

Figure S9. The depletion of $CD3^{+}$ T cells in mice upon the aCD3 antibody administration.

Representative flow cytometric images from at least 3 independent mice showing the depletion of $CD3^{+}$ T cells in the MLN and LP of $p53^{+/+}$ mice at 6 days upon IgG or the aCD3 antibody (200 µg/mouse, once) treatment.

Supplementary Figure 10

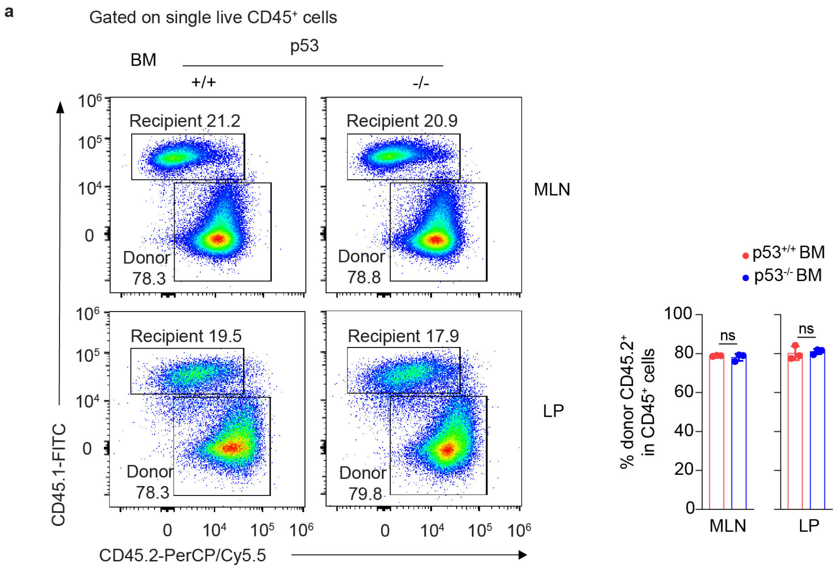

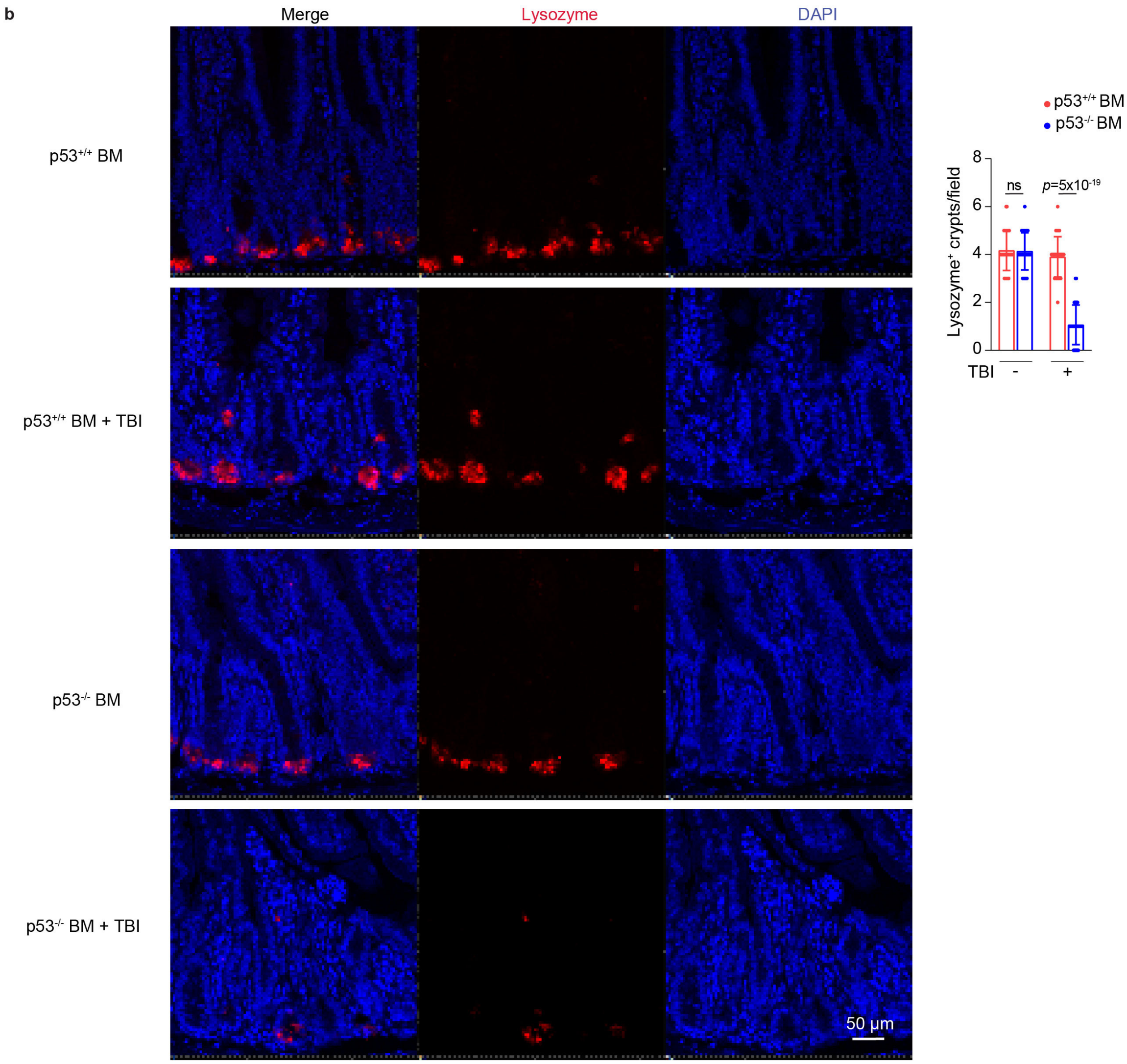

Figure S10. Adoptive transfer of the BM derived from p53$^{-/-}$ mice into p53$^{+/+}$ mice exacerbates the damage of Paneth cells post-TBI.

a. CD45.1^{+} mice were adoptively transferred with BM cells isolated from CD45.2^{+} p53$^{+/+}$ and p53$^{-/-}$ mice. The reconstitution of donor CD45.2^{+} CD45^{+} immune cells in the MLN and LP was examined at 28 days after adoptive transfer by flow cytometric assays. n = 3 mice/group. **b**. Representative images from at least 3 independent mice (left panels) and quantification (right panel) of IF staining of the lysozyme in the SI of mice at 5 days post 12 Gy TBI. n = 30 crypts from at least 3 mice/group. Data are presented as mean ± SD from at least 3 independent experiments. ns: not significant, two-tailed Student's *t*-test. Source data are provided as a Source Data file.

Supplementary Figure 11

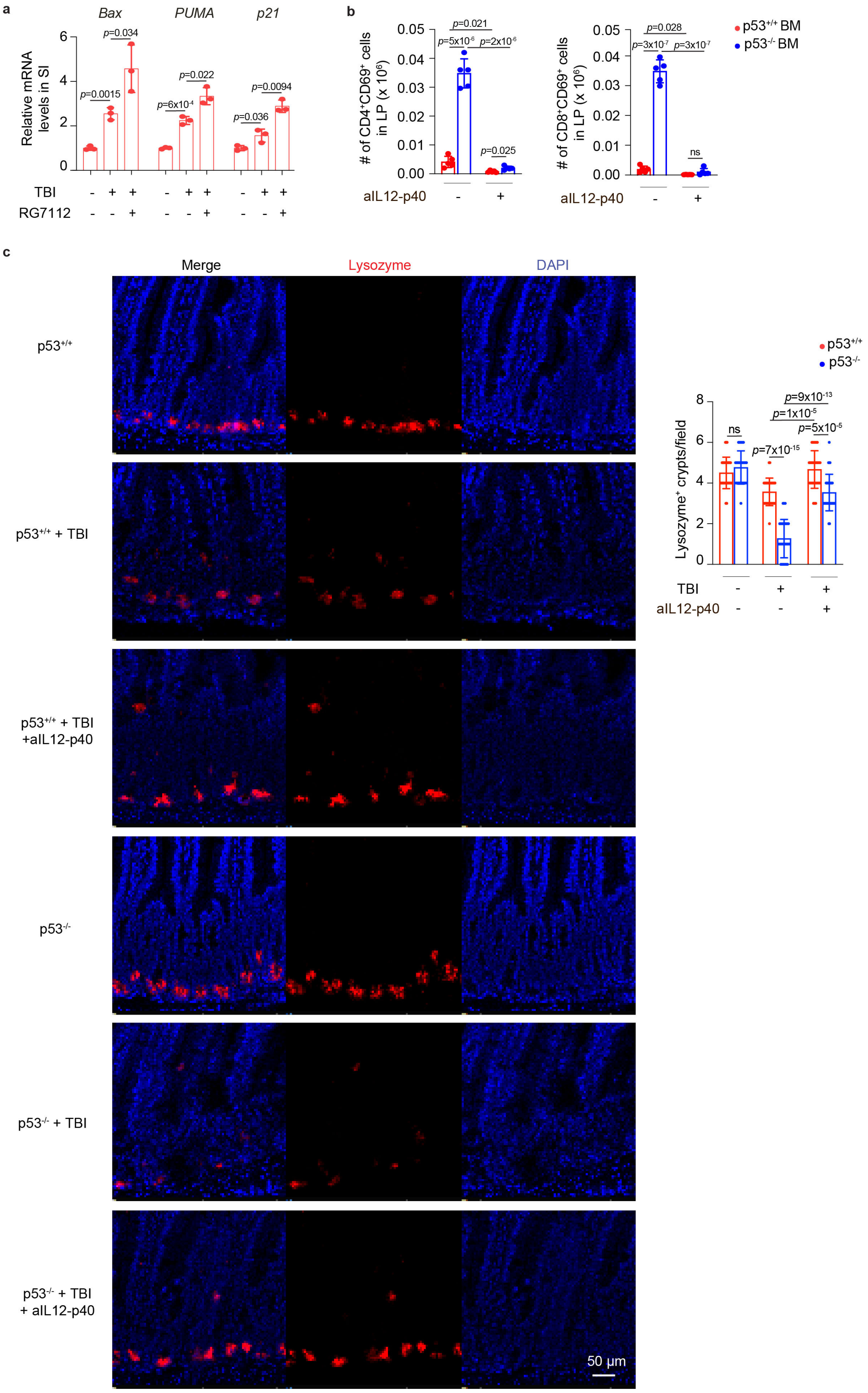

Figure S11. aIL12-p40 antibody reduces the damage of Paneth cells in the SI of mice post-TBI.

a. The mRNA levels of *BAX*, *PUMA* and *p21* in the SI of $p53^{+/+}$ mice at 6 hours post 12 Gy TBI along with or without RG7112 treatment (*i.p.*, 50 μg/g body weight, twice, 8 hours before and 1 hour post-TBI) determined by qPCR assays. n = 3 mice/group. **b**. The number of $CD69^+$ activated $CD4^+$ (left panel) and $CD8^+$ (right panel) T cells in the LP of mice at 3 days post-TBI determined by flow cytometric assays. n = 5 mice/group. **c**. Representative images from at least 3 independent mice (left panels) and quantification (right panel) of IF staining of lysozyme in the SI of mice at 3 days post-TBI. n = 30 crypts from at least 3 mice/group. Data are presented as mean ± SD from at least 3 independent experiments. ns: not significant, two-tailed Student's *t*-test followed by Bonferroni correction. Source data are provided as a Source Data file.

Supplementary Figure 12

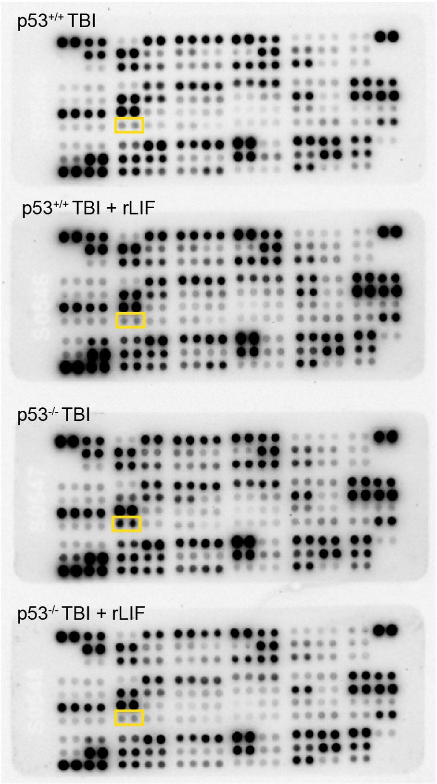

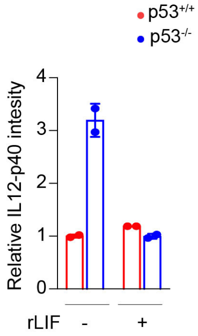

Figure S12. rLIF administration inhibits the IL12-p40 production in p53$^{-/-}$ mice post-TBI. Left: representative images of cytokine levels in the serum from p53$^{+/+}$ and p53$^{-/-}$ mice with or without rLIF administration at 3 days post 12 Gy TBI detected by using the Proteome Profiler Mouse XL Cytokine Array. Right: quantification of IL12-p40 levels in Cytokine Array. Each array represents a pool of 3 mice with the same treatment. Dots in yellow box represent IL12-p40. Source data are provided as a Source Data file.

Supplementary Figure 13

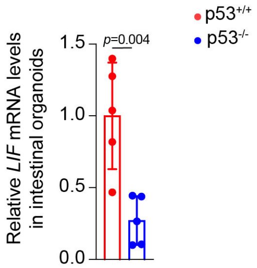

Figure S13. p53 deficiency reduces LIF expression levels in intestinal organoids.

The mRNA levels of *LIF* in intestinal organoids derived from $p53^{+/+}$ and $p53^{-/-}$ mice determined by qPCR assays. n = 5/group. Data are presented as mean ± SD from at least 3 independent experiments. Two-tailed Student's *t*-test. Source data are provided as a Source Data file.

Supplementary Figure 14

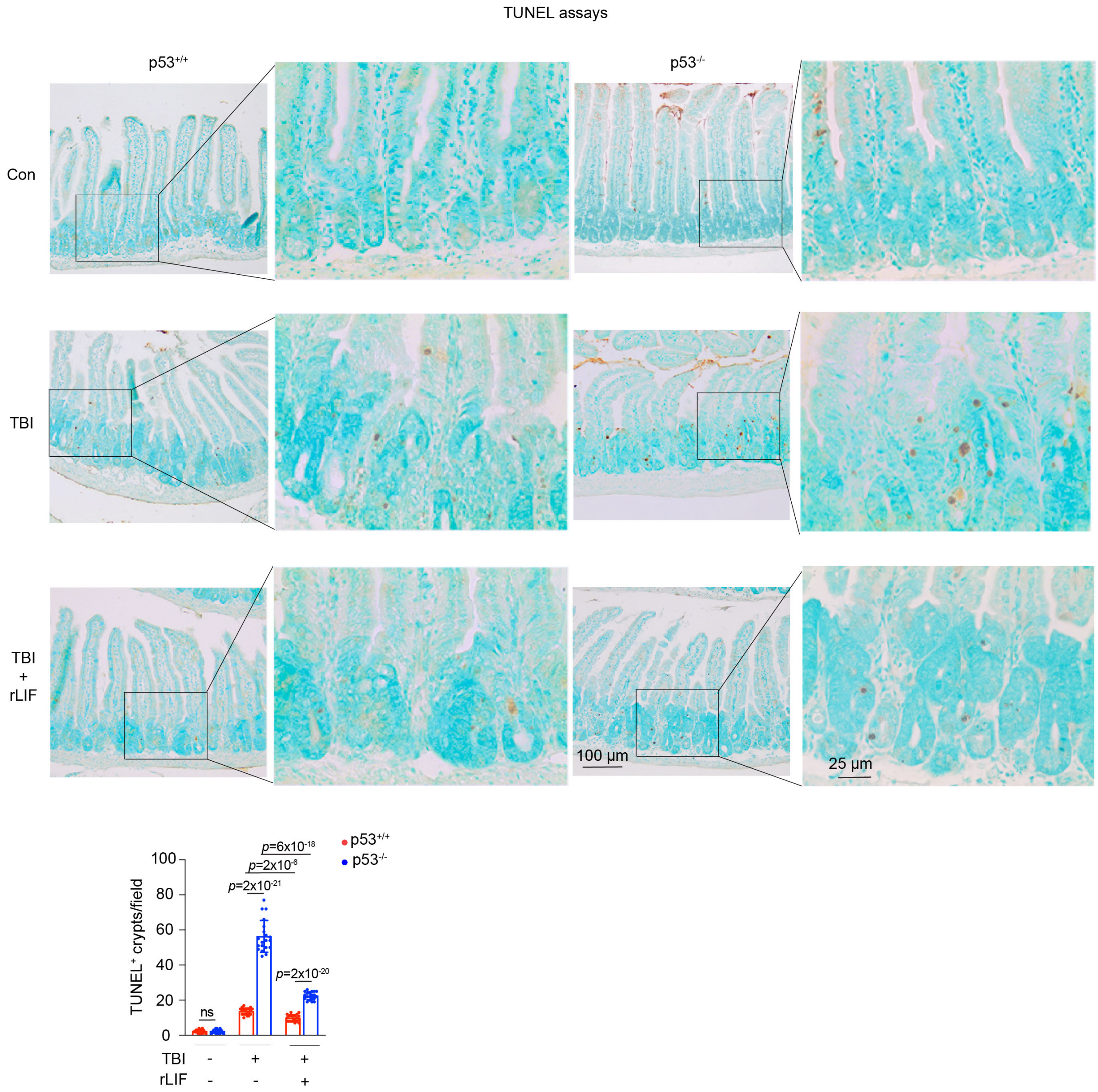

Figure S14. rLIF administration reduces cell death in the SI in p53$^{-/-}$ mice post-TBI. Representative images from at least 3 independent mice (upper panels) and quantification (bottom panel) of TUNEL assays in the SI of p53$^{+/+}$ and p53$^{-/-}$ mice with or without rLIF administration at 3 days post 12 Gy TBI. n = 20 fields from at least 3 mice/group. Data are presented as mean ± SD from at least 3 independent experiments. ns: not significant, two-tailed Student's *t*-test followed by Bonferroni correction. Source data are provided as a Source Data file.

Supplementary Figure 15

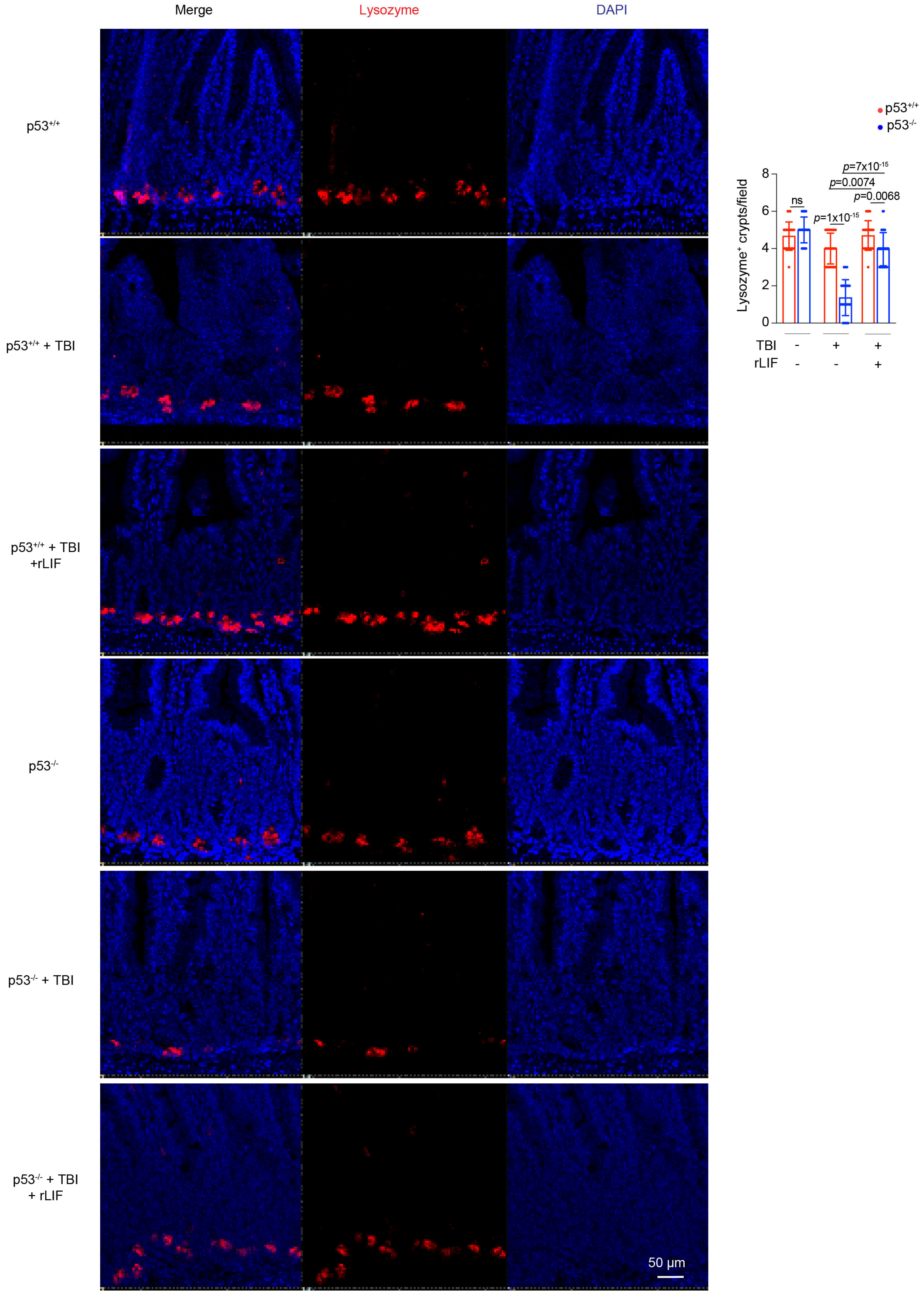

Figure S15. rLIF administration reduces the damage of Paneth cells in p53$^{-/-}$ mice post-TBI. Representative images from at least 3 independent mice (left panels) and quantification (right panel) of IF staining of lysozyme in the SI of mice at 3 days post-TBI. n = 30 crypts from at least 3 mice/group. Data are presented as mean ± SD from at least 3 independent experiments. ns: not significant, two-tailed Student's *t*-test followed by Bonferroni correction. Source data are provided as a Source Data file.

Supplementary Table S1. Information of flow antibodies used in this study

Flow antibodies and reagents			
REAGENT or RESOURCE	SOURCE	Cata#	CLONE
eFluor450 anti-mouse CD4	eBioscience	48-0042-82	RM4-5
Brilliant Violet 510™ anti-mouse CX3CR1	Biolegend	149025	SA011F11
Super Bright 702 anti-mouse CD45	Thermo Fisher	67-0451-82	30-F11
FITC anti-mouse Ep-CAM	Biolegend	118208	G8.8
FITC anti-mouse CD45.1	Biolegend	110706	A20
Per/Cy5.5 anti-mouse CD45.2	Biolegend	109828	104
PE anti-mouse CD11c	Biolegend	117308	N418
PE anti-mouse CD8	Biolegend	100708	53-6.7
PE/Cy7 anti-mouse MHC-II	Biolegend	107630	M5/114.15.2
APC anti-mouse CD3	Biolegend	100236	17A2
PE/Cy7 anti-mouse CD25	BD	552880	PC61
Alex Fluor 700 anti-mouse CD69	Biolegend	104539	H1.2F3
Alex Fluor 700 anti-mouse CD11b	Biolegend	101222	M1/70
Alex Fluor 700 anti-mouse TNFα	Biolegend	506338	MP6-XT22
Cell Stimulation Cocktail	Thermo Scientific	00-4975-03	N/A
BD cytofix/cytoperm Fixation/Permeabilization Solution Kit	BD	554714	N/A
Ultra-LEAF™ Purified anti-mouse CD16/32	Biolegend	101339	93
LIVE/DEAD™ Fixable Yellow Dead Cell Stain Kit	Thermo Fisher	L34968	N/A
Abc Total Antibody Compensation Bead Kit	Thermo Fisher	A10497	N/A

Supplementary Table S2. Sequences of qPCR primers

Gene	Forward Primer	Reverse Primer
β-actin	5’ GAACCCTAAGGCCAACCGTGAAAAGATGAC 3’	5’ GCAGGATGGCGTGAGGGAGAGCA 3’
TNFα	5’ CTGAACTTCGGGGTGATCGG 3’	5’ GGCTTGTCACTCGAATTTTGAGA 3’
BAX	5’ TGAAGACAGGGGCCTTTTTG 3’	5’ AATTCGCCGGAGACACTCG 3’
PUMA	5’ GTCGCTACCGTCGTGACTTC 3’	5’ CAGACATGCACCTACCCAGC 3’
p21	5’ CCTGGTGATGTCCGACCTG 3’	5’ CCATGAGCGCATCGCAATC 3’
