# Supplementary material for: p53 suppresses MHC class II presentation by intestinal epithelium to protect against radiation-induced gastrointestinal syndrome
Source: Nat Commun. 2024 Jan 2;15:137. doi: 10.1038/s41467-023-44390-w (PMC10762193; doi:10.1038/s41467-023-44390-w)
Supplement: Supplementary file 3 — Reporting Summary [file 41467_2023_44390_MOESM3_ESM.pdf]

nature portfolio

Corresponding author(s): Wenwei Hu and Zhaohui Feng
Last updated by author(s): Nov 20, 2023

Reporting Summary

Nature Portfolio wishes to improve the reproducibility of the work that we publish. This form provides structure for consistency and transparency in reporting. For further information on Nature Portfolio policies, see our Editorial Policies and the Editorial Policy Checklist.

Statistics

For all statistical analyses, confirm that the following items are present in the figure legend, table legend, main text, or Methods section.

n/a	Confirmed	
☐	☒	The exact sample size (*n*) for each experimental group/condition, given as a discrete number and unit of measurement
☐	☒	A statement on whether measurements were taken from distinct samples or whether the same sample was measured repeatedly
☐	☒	The statistical test(s) used AND whether they are one- or two-sided<br>*Only common tests should be described solely by name; describe more complex techniques in the Methods section.*
☒	☐	A description of all covariates tested
☐	☒	A description of any assumptions or corrections, such as tests of normality and adjustment for multiple comparisons
☐	☒	A full description of the statistical parameters including central tendency (e.g. means) or other basic estimates (e.g. regression coefficient) AND variation (e.g. standard deviation) or associated estimates of uncertainty (e.g. confidence intervals)
☐	☒	For null hypothesis testing, the test statistic (e.g. *F*, *t*, *r*) with confidence intervals, effect sizes, degrees of freedom and *P* value noted<br>*Give P values as exact values whenever suitable.*
☒	☐	For Bayesian analysis, information on the choice of priors and Markov chain Monte Carlo settings
☒	☐	For hierarchical and complex designs, identification of the appropriate level for tests and full reporting of outcomes
☒	☐	Estimates of effect sizes (e.g. Cohen's *d*, Pearson's *r*), indicating how they were calculated

Our web collection on statistics for biologists contains articles on many of the points above.

Software and code

Policy information about availability of computer code

Data collection	Nikon A1R-Si Confocal Microscope, Attune NxT Flow Cytometer
Data analysis	FlowJo_v10.8.0, GraphPad Prism 9

For manuscripts utilizing custom algorithms or software that are central to the research but not yet described in published literature, software must be made available to editors and reviewers. We strongly encourage code deposition in a community repository (e.g. GitHub). See the Nature Portfolio guidelines for submitting code & software for further information.

Data

Policy information about availability of data

All manuscripts must include a data availability statement. This statement should provide the following information, where applicable:

- Accession codes, unique identifiers, or web links for publicly available datasets
- A description of any restrictions on data availability
- For clinical datasets or third party data, please ensure that the statement adheres to our policy

Source data are provided with this paper. The RNA-seq data generated in this study have been deposited in the Gene Expression Omnibus (GEO) database under accession number code GSE226421. (https://www.ncbi.nlm.nih.gov/geo/query/acc.cgi?acc=GSE226421).

Research involving human participants, their data, or biological material

Policy information about studies with human participants or human data. See also policy information about sex, gender (identity/presentation), and sexual orientation and race, ethnicity and racism.

Reporting on sex and gender	NA
Reporting on race, ethnicity, or other socially relevant groupings	NA
Population characteristics	NA
Recruitment	NA
Ethics oversight	NA

Note that full information on the approval of the study protocol must also be provided in the manuscript.

Field-specific reporting

Please select the one below that is the best fit for your research. If you are not sure, read the appropriate sections before making your selection.

[x] Life sciences [ ] Behavioural & social sciences [ ] Ecological, evolutionary & environmental sciences

For a reference copy of the document with all sections, see nature.com/documents/nr-reporting-summary-flat.pdf

Life sciences study design

All studies must disclose on these points even when the disclosure is negative.

Sample size	Sample sizes were chosen based on the power calculation.
Data exclusions	No data exclusions.
Replication	Experimental finding were replicated with at least 3 independent biological repeats.
Randomization	Animals and organoids were randomly assigned to different treatment groups.
Blinding	The investigators were blinded to the group allocation during experiments and when assessing outcomes.

Reporting for specific materials, systems and methods

We require information from authors about some types of materials, experimental systems and methods used in many studies. Here, indicate whether each material, system or method listed is relevant to your study. If you are not sure if a list item applies to your research, read the appropriate section before selecting a response.

Materials & experimental systems

n/a	Involved in the study	
[ ]	[x]	Antibodies
[x]	[ ]	Eukaryotic cell lines
[x]	[ ]	Palaeontology and archaeology
[ ]	[x]	Animals and other organisms
[x]	[ ]	Clinical data
[x]	[ ]	Dual use research of concern
[x]	[ ]	Plants

Methods

n/a	Involved in the study	
[x]	[ ]	ChIP-seq
[ ]	[x]	Flow cytometry
[x]	[ ]	MRI-based neuroimaging

Antibodies

Antibodies used	Goat Anti-Rabbit IgG (H+L), Biotinylated (Cata#: BA-1000-1.5, Vector Laboratories)<br>Goat Anti-Rabbit IgG (H+L), Cy5 ® (Cata#: ab6564, Abcam)<br>Goat Anti-Rabbit IgG (H+L), Alexa Fluor™ 555 (Cata#: A-21428, Invitrogen)<br>Goat Anti-Rat IgG (H+L), Alexa Fluor™ 555 (Cata#: ab150158, Abcam)<br>aIL12-p40 antibody (Cata#: 505309, Biolegend)<br>aCD3 antibody (Cata#: BE0001-1, Bio X Cell)

anti-Olfm4 antibody (Cata#: 39141S, Cell Signaling)
anti-lysozyme antibody(Cata#: ab108508, Abcam)
anti-CD45 antibody(Cata#: 550539, BD)
anti-CD3 antibody(Cata#: 99940, Cell Signaling)
anti-MHC-II- Alexa Fluor® 594 (Cata#: 107650, Biolegend)
eFluor450 anti-mouse CD4 eBioscience 48-0042-82
Brilliant Violet 510™ anti-mouse CX3CR1 Biolegend 149025
Super Bright 702 anti-mouse CD45 Thermo Fisher 67-0451-82
FITC anti-mouse Ep-CAM Biolegend 118208
FITC anti-mouse CD45.1 Biolegend 110706
Per/Cy5.5 anti-mouse CD45.2 Biolegend 109828
PE anti-mouse CD11c Biolegend 117308
PE anti-mouse CD8 Biolegend 100708
PE/Cy7 anti-mouse MHC-II Biolegend 107630
APC anti-mouse CD3 Biolegend 100236
PE/Cy7 anti-mouse CD25 BD 552880
Alex Fluor 700 anti-mouse CD69 Biolegend 104539
Alex Fluor 700 anti-mouse CD11b Biolegend 101222
Alex Fluor 700 anti-mouse TNFα Biolegend 506338

Validation

Validation of all commercial antibodies are available at the manufacturer's website. The reference for each antibody is listed below:
Goat Anti-Rabbit IgG (H+L), Biotinylated (Cata#: BA-1000-1.5, Vector Laboratories)
https://vectorlabs.com/products/biotinylated-goat-anti-rabbit-igg/
Goat Anti-Rabbit IgG (H+L), Cy5 ® (Cata#: ab6564, Abcam)
https://www.abcam.com/products/secondary-antibodies/goat-rabbit-igg-hl-cy5--preadsorbed-ab6564.html
Goat Anti-Rabbit IgG (H+L), Alexa Fluor™ 555 (Cata#: A-21428, Invitrogen)
https://www.thermofisher.com/antibody/product/Goat-anti-Rabbit-IgG-H-L-Cross-Adsorbed-Secondary-Antibody-Polyclonal/A-21428
Goat Anti-Rat IgG (H+L), Alexa Fluor™ 555 (Cata#: ab150158, Abcam)
https://www.abcam.com/products/secondary-antibodies/goat-rat-igg-hl-alexa-fluor-555-ab150158.html
aIL12-p40 antibody (Cata#: 505309, Biolegend)
https://www.biolegend.com/fr-lu/products/ultra-leaf-low-endotoxin-azide-free-purified-anti-mouse-il-12-il-23-p40-monomer-dimer-heterodimer-antibody-7751
aCD3 antibody (Cata#: BE0001-1, Bio X Cell)
https://bioxcell.com/invivomab-anti-mouse-cd3e-be0001-1
anti-Olfm4 antibody (Cata#: 39141S, Cell Signaling)
https://www.cellsignal.com/products/primary-antibodies/olfm4-d6y5a-xp-rabbit-mab-mouse-specific/39141?_requestid=3137419
anti-lysozyme antibody(Cata#: ab108508, Abcam)
https://www.abcam.com/products/primary-antibodies/lysozyme-antibody-epr29942-ab108508.html
anti-CD45 antibody(Cata#: 550539, BD)
https://www.bdbiosciences.com/en-us/products/reagents/microscopy-imaging-reagents/immunohistochemistry-reagents/purified-rat-anti-mouse-cd45.550539
anti-CD3 antibody(Cata#: 99940, Cell Signaling)
https://www.cellsignal.com/products/primary-antibodies/cd3e-d4v8l-rabbit-mab/99940
anti-MHC-II- Alexa Fluor® 594 (Cata#: 107650, Biolegend)
https://www.biolegend.com/de-de/products/alexa-fluor-594-anti-mouse-i-a-i-e-antibody-12448?GroupID=BLG4736
eFluor450 anti-mouse CD4 eBioscience 48-0042-82
https://www.thermofisher.com/antibody/product/CD4-Antibody-clone-RM4-5-Monoclonal/48-0042-82
Brilliant Violet 510™ anti-mouse CX3CR1 Biolegend 149025
https://www.biolegend.com/de-at/products/brilliant-violet-510-anti-mouse-cx3cr1-antibody-11853
Super Bright 702 anti-mouse CD45 Thermo Fisher 67-0451-82
https://www.thermofisher.com/antibody/product/CD45-Antibody-clone-30-F11-Monoclonal/67-0451-82
FITC anti-mouse Ep-CAM Biolegend 118208
https://www.biolegend.com/fr-ch/products/fitc-anti-mouse-cd326-ep-cam-antibody-4971?GroupID=BLG5748
FITC anti-mouse CD45.1 Biolegend 110706
https://www.biolegend.com/de-de/products/fitc-anti-mouse-cd45-1-antibody-198?GroupID=BLG1933
Per/Cy5.5 anti-mouse CD45.2 Biolegend 109828
https://www.biolegend.com/ja-jp/products/percp-cyanine5-5-anti-mouse-cd452-antibody-4271
PE anti-mouse CD11c Biolegend 117308
https://www.biolegend.com/en-us/products/pe-anti-mouse-cd11c-antibody-1816?GroupID=BLG11937
PE anti-mouse CD8 Biolegend 100708
https://www.biolegend.com/fr-ch/products/pe-anti-mouse-cd8a-antibody-155?GroupID=BLG2559
PE/Cy7 anti-mouse MHC-II Biolegend 107630
https://www.biolegend.com/en-us/products/pe-cyanine7-anti-mouse-i-a-i-e-antibody-6136?GroupID=BLG11931
APC anti-mouse CD3 Biolegend 100236
https://www.biolegend.com/en-us/products/apc-anti-mouse-cd3-antibody-8055?GroupID=BLG242
PE/Cy7 anti-mouse CD25 BD 552880
https://www.bdbiosciences.com/en-us/products/reagents/flow-cytometry-reagents/research-reagents/single-color-antibodies-ruo/pe-cy-7-rat-anti-mouse-cd25.561780

Alex Fluor 700 anti-mouse CD69 Biolegend 104539
https://www.biolegend.com/en-us/punchout/search-results/alexa-fluor-700-anti-mouse-cd69-antibody-12352
Alex Fluor 700 anti-mouse CD11b Biolegend 101222
https://www.biolegend.com/fr-ch/products/alexa-fluor-700-anti-mouse-human-cd11b-antibody-3388?GroupID=BLG10599
Alex Fluor 700 anti-mouse TNFα Biolegend 506338
https://www.biolegend.com/fr-fr/products/alexa-fluor-700-anti-mouse-tnf-alpha-antibody-9146?GroupID=GROUP24

Animals and other research organisms

Policy information about studies involving animals; ARRIVE guidelines recommended for reporting animal research, and Sex and Gender in Research

Laboratory animals	WT p53 (p53+/+, Cata#: 000664), p53-deficient (p53-/-, Cata#: 002101), CD45.1 (Cata#: 002014) and ApcMin/+ (Cata#: 002020) mice were obtained from the Jackson Laboratory. IL12-p40-IRES-eYFP mice were a kind gift from Dr. Timothy E. O'Sullivan at UCLA. Mice were housed under a 12-h light/dark cycle with 6 am on–6 pm off. The temperature is maintained between 70° and 74 °F and the humidity is between 30 and 70%. Age- and gender-matched mice at 8-12-week-old were used for experiments in this study.
Wild animals	No wild animal was involved in this study.
Reporting on sex	Not indicated.
Field-collected samples	Field-collected samples were not involved in this study.
Ethics oversight	All mouse experiments were approved by the Institutional Animal Care and Use Committee (IACUC) of Rutgers University.

Note that full information on the approval of the study protocol must also be provided in the manuscript.

Flow Cytometry

Plots

Confirm that:

- [x] The axis labels state the marker and fluorochrome used (e.g. CD4-FITC).
- [x] The axis scales are clearly visible. Include numbers along axes only for bottom left plot of group (a 'group' is an analysis of identical markers).
- [x] All plots are contour plots with outliers or pseudocolor plots.
- [x] A numerical value for number of cells or percentage (with statistics) is provided.

Methodology

Sample preparation	The spleen and MLN were mashed through a 70 μm cell strainer. Red blood cells were removed by lysing in RBC buffer (Cata#: 420302, BioLegend). The LP and EPI single-cell suspensions were prepared by using a Lamina Propria Dissociation Kit (Cata#: 130-097-410, Miltenyi Biotec) and a gentleMACSTM Dissociator (Cata#: 130-093-235, Miltenyi Biotec).
Instrument	Attune NxT Flow Cytometer
Software	FlowJo_v10.8.0
Cell population abundance	Cell population abundance were visualized by the use of pseudocolor dot plots including outliers.
Gating strategy	In all experiments, populations were gated on FSC/SSC. Dead cells were excluded by using LIVE/DEAD™ Fixable Yellow Dead Cell Stain Kit. Cells populations were identified as described in Figures or Figure legends. Positive and negative cells were identified based on clear boundaries between the two populations.

- [x] Tick this box to confirm that a figure exemplifying the gating strategy is provided in the Supplementary Information.
